# Behind the Genetics: The Role of Epigenetics in Infertility-Related Testicular Dysfunction

**DOI:** 10.3390/life14070803

**Published:** 2024-06-26

**Authors:** Andrea Crafa, Rossella Cannarella, Aldo E. Calogero, Sezgin Gunes, Ashok Agarwal

**Affiliations:** 1 Department of Clinical and Experimental Medicine, University of Catania, 95123 Catania, Italy; crafa.andrea@outlook.it (A.C.); rosella.cannarella@phd.unict.it (R.C.); aldo.calogero@unict.it (A.E.C.); 2 Global Andrology Forum, Moreland Hills, OH 44022, USA; 3 Glickman Urological & Kidney Institute, Cleveland Clinic Foundation, Cleveland, OH 44106, USA; 4 Department of Medical Biology, Faculty of Medicine, Ondokuz Mayis University, 55280 Samsun, Türkiye; 5 Cleveland Clinic Foundation, Cleveland, OH 44195, USA

**Keywords:** male infertility, epigenetics, varicocele, obesity, risky lifestyle behaviors, sperm DNA methylation, sperm histone modifications, sperm non-coding RNA

## Abstract

In recent decades, we have witnessed a progressive decline in male fertility. This is partly related to the increased prevalence of chronic diseases (e.g., obesity and diabetes mellitus) and risky lifestyle behaviors. These conditions alter male fertility through various non-genetic mechanisms. However, there is increasing evidence that they are also capable of causing sperm epigenetic alterations, which, in turn, can cause infertility. Furthermore, these modifications could be transmitted to offspring, altering their general and reproductive health. Therefore, these epigenetic modifications could represent one of the causes of the progressive decline in sperm count recorded in recent decades. This review focuses on highlighting epigenetic modifications at the sperm level induced by non-genetic causes of infertility. In detail, the effects on DNA methylation, histone modifications, and the expression profiles of non-coding RNAs are evaluated. Finally, a focus on the risk of transgenerational inheritance is presented. Our narrative review aims to demonstrate how certain conditions can alter gene expression, potentially leading to the transmission of anomalies to future generations. It emphasizes the importance of the early detection and treatment of reversible conditions (such as obesity and varicocele) and the modification of risky lifestyle behaviors. Addressing these issues is crucial for individual health, in preserving fertility, and in ensuring the well-being of future generations.

## 1. Introduction

Male infertility is one of the main topics studied to date in andrology. Furthermore, genetic studies are the most representative in this field [1]. The in-depth study of genetic aspects is probably attributable to the still high rate of patients diagnosed with idiopathic infertility, which, in some studies, has been estimated to be as high as 72% [2].

To date, a genetic cause of male infertility appears to affect approximately 15% of male infertile patients. This prevalence reaches 25% if we consider patients with azoospermia, indicating that the role of genetics is more significant in cases of more severe alterations in sperm parameters [3]. Consequently, evidence from the literature is leading to the identification of a growing number of genes whose mutations could be involved in spermatogenetic failure, suggesting the adoption of genetic panels to be researched to reduce the rate of patients with idiopathic infertility [4].

More recently, epigenetic changes have also been associated with several male infertility phenotypes. The main epigenetic modifications investigated are altered sperm DNA methylation (globally and at the level of imprinted genes and other specific genes), histone modifications, and the expression profiles of non-coding RNAs [5]. For example, the hypomethylation of the maternally imprinted *H19* gene, resulting in increased expression, is associated with infertility and, in particular, oligozoospermia or recurrent pregnancy loss [6].

In addition to genetics, many other conditions are associated with alterations in male fertility. For example, varicocele can have deleterious effects on the testicular function of some patients, compromising semen quality and sperm function, resulting in a low rate of reproductive success [7]. Furthermore, over the past 50 years, we have seen a gradual decline in male fertility potential, with a 51.6% reduction in sperm concentrations between 1973 and 2018 [8]. The reason for this decline is not entirely apparent. Still, numerous factors come into play, including the increase in the prevalence of chronic diseases, such as obesity and diabetes mellitus, and the increase in exposure to toxic substances (use of medications, cigarette smoking, and alcohol consumption) [9]. There are several non-genetic mechanisms by which these factors can cause infertility by altering spermatogenesis. However, it is equally clear that exposure to harmful lifestyle factors, such as an unbalanced diet, obesity, poor physical activity, tobacco smoking, alcohol consumption, environmental pollutants, and psychological stress, can alter epigenetic patterns [10]. This suggests that the alterations of gene expression profiles caused by epigenetic changes could be another mechanism through which these factors can influence male reproductive function. This aspect is fundamental, considering that epigenetic modifications could be transmitted to offspring, possibly affecting their health [11].

Therefore, this review analyzes epigenetic changes among the main non-genetic causes of testicular dysfunction leading to infertility. The novelty of the review stems from the fact that it examines, in a comprehensive and structured manner, the role of epigenetic modifications in various non-genetic causes of male infertility, focusing, in particular, on how these modifications can impair male fertility. This is new, as many reviews focus only on the inheritance of these changes. Particular attention is paid to varicocele, obesity, physical activity, eating habits, stress, and other harmful lifestyles (smoking, alcohol, substance abuse, exposure to endocrine disruptors). Finally, the risk of transgenerational inheritance is also reviewed.

## 2. Varicocele and Epigenetic Modifications

Among the epigenetic changes associated with varicocele is the reduction in the global methylation pattern of sperm DNA. Notably, a study comparing a small number of varicocele patients and fertile men showed reduced global sperm DNA methylation in varicocele patients [12]. Furthermore, 1695 statistically significantly differentially methylated regions (DMRs) in the varicocele group compared with the controls were found. In a gene ontology analysis, these DMRs were related to genes involved in gamete generation, piRNA metabolic processes, the meiotic cell cycle, and male meiosis [12]. In addition, in the mouse, these regions are associated with genes involved in an abnormal spermatocyte morphology and azoospermia [12]. Finally, 443 of these regions also overlapped with those identified as related to poor sperm motility and viability in a previous study [12,13]. Another study observed that the global hypomethylation of sperm DNA in patients with varicocele was positively correlated with the increased rate of sperm DNA fragmentation (SDF) occurring in these patients. This correlation was independent of the oxidative stress levels [14]. The results of these studies show the role of sperm DNA methylation modification as an independent factor associated with infertility in patients with varicocele [12,14].

A study showing that varicocele repair is associated with an increase in global sperm DNA methylation compared to baseline further confirms the role of varicocele in inducing sperm DNA hypomethylation, although this improvement was statistically significant only in patients with associated oligozoospermia [15].

An explanation of the mechanisms underlying this global hypomethylation of sperm DNA was provided by a study showing the increased expression of DNA methyl-transferases (DNMTs) 3A and 3B in patients with varicocele. The authors hypothesized that these enzymes acquire demethylating activity following the increase in oxidative stress induced by varicocele, causing hypomethylation [16]. A study showed that another possible mechanism of the observed hypomethylation could be the overexpression of the ten-eleven translocation 2 (TET2) protein in the rodent varicocele model, which, in turn, has demethylase activity [17].

There is no evidence that the presence of varicocele plays a role in the methylation defects of imprinted genes. Indeed, a study on rats with varicocele showed no difference in the methylation rate of the *H19* and small nuclear ribonucleoprotein polypeptide N (*SNRPN*) genes compared to control rats [18]. No previous study has investigated the correlation between varicocele and histone modifications. However, patients with varicocele appear to have a protamination defect that would promote damage to sperm DNA and exposure to possible DNA epigenetic modifications [19].

In animal and human studies, the role of non-coding RNAs in the pathogenesis of damage induced by varicocele is investigated to a greater extent. A study showed the upregulation of rno-miR-210-3p, rno-miR-6316, rno-miR-190a-5p, and rno-miR-135b-5p in rats with varicocele compared with healthy and sham-operated rats. The authors also showed that these miRNAs are involved in the expression of genes that regulate the immune response and the apoptotic process, suggesting that the dysregulation of these mechanisms could underlie varicocele-induced sperm damage [20].

The role of miR-210-3p in the pathogenesis of varicocele-induced damage has also been reported in human studies. Xu and colleagues showed the approximately 2.18-fold higher expression of this miRNA in patients with varicocele compared to controls. Moreover, the greater the expression of miR-210-3p, the higher the degree of varicocele, suggesting a correlation between the expression levels of this miRNA and the severity of varicocele. Finally, the miRNA levels were an independent predictor of sperm parameter abnormalities, suggesting their use as a possible early biomarker of varicocele-induced sperm damage, helping to decide which patients should undergo varicocele repair to preserve their reproductive function [21]. Another study confirmed these findings, highlighting a negative correlation between the miR-210-3p levels and serum inhibin B levels [22]. The latter is highly sensitive and specific to spermatogenesis and shows a positive correlation with a better sperm count and testicular volume and more promising results from testicular biopsies [23]. Moreover, since inhibin B is directly secreted by Sertoli cells, reflecting their function, this study suggests that miR-210-3p expression may also be used as an early marker of damage to Sertoli cells induced by varicocele [22]. The increased expression of this miRNA is directly responsible for varicocele-induced damage, as demonstrated by a study that prospectively evaluated patients with varicocele and normal sperm parameters at enrollment. After two years of follow-up, the patients were divided into two groups depending on whether or not they developed abnormalities in sperm parameters. Significantly higher levels of miR-210-3p were present in patients who developed abnormalities in sperm parameters than in patients who did not develop abnormalities. Furthermore, in patients with higher levels of the miRNA, a higher rate of cleaved (and therefore activated) caspase-3 was found, suggesting that the mechanism of action of miR-210-3p-induced damage involves the greater activation of caspase-3, which in turn is related to apoptotic processes [24]. 

The expression profiles of other miRNAs have also been evaluated in infertile patients with varicocele [25,26]. For example, Mostafa and colleagues observed that patients with varicocele and oligoasthenoteratozoospermia (OAT) had reduced seminal expression levels of miRNA-122, miRNA-181a, and miRNA-34c5 compared to patients with OAT but without varicocele and to fertile men with varicocele. Further strengthening the prognostic role, the authors observed a positive correlation between the expression of these miRNAs and the sperm concentration, total sperm motility, a normal sperm morphology, seminal glutathione peroxidase, and seminal antiapoptotic B-cell lymphoma 2 (BCL2) protein. They also showed a negative correlation between seminal malondialdehyde and seminal pro-apoptotic BCL-2-associated X-protein (BAX). These results confirm the role of miRNAs in regulating apoptotic processes and generating oxidative damage [25]. Varicocele patients also show the downregulation of miR-15a, which usually inhibits the translation of the heat shock protein A1B (HSPA1B) messenger, involved in antiapoptotic processes. Therefore, in a patient with varicocele, HSPA1B was higher than in the control group. The increase in HSPA1B appears to be a defense mechanism against varicocele-induced oxidative stress damage. Once again, this study highlights the role of miRNAs in regulating these processes [27]. Finally, miRNA expression has also been questioned as a marker to predict the appearance of spermatozoa in the ejaculates of patients with non-obstructive azoospermia (NOA) affected by varicocele. Notably, a study showed that, in patients with NOA who had no spermatozoa in the ejaculate after varicocele repair, the miR-192a expression levels were significantly higher than those in patients who experienced the appearance of spermatozoa. These results suggest that miR-192a could be used as a biomarker to select patients with NOA and varicocele to undergo varicocele repair [28].

Regarding long non-coding RNAs (lncRNAs), it has been observed that these are also related to the pathogenesis of oxidative stress damage induced by varicocele. A study in mouse models showed the presence of 10 lncRNAs differentially expressed in rats with varicocele compared to those without varicocele. In detail, these lncRNAs appear to be involved in the regulation of the phosphatidylinositol 3-kinase-protein kinase B signaling pathway, responsible for promoting germ cell apoptosis and inhibiting the proliferation of spermatogonia, spermatocyte proliferation, and spermatocyte meiosis [29]. Ata-Abadi and colleagues reported the overexpression of the hypoxia-related lncRNAs miR-210HG and MLLT4-AS1 in the ejaculated spermatozoa of infertile patients with varicocele. These lncRNAs are negatively correlated with the sperm count and motility and positively with the oxygen free radical levels, further highlighting their pathogenetic role [30]. Accordingly, another study involving 188 patients with varicocele showed the increased expression of lncRNA MIR210HG in patients compared to healthy controls. Furthermore, the expression levels were higher with increasing varicocele severity. Moreover, the varicocele patients with higher miR-210HG levels were also those who more frequently had sperm parameter abnormalities. An analysis using the receiver operating characteristic (ROC) curve showed that the miR-210HG levels could predict, independently of other factors, the presence of sperm parameter abnormalities, with sensitivity of 87.1% and specificity of 82.1%. Therefore, this study seems to confirm that the increased expression of miR-210HG correlates with the severity of varicocele and the sperm damage that it causes. The authors hypothesize that the pathogenetic mechanism of this damage might be based on the ability of this lncRNA to bind the hypoxia-inducible factor 1α (HIF-1α) messenger, promoting its translation. HIF1-α would, therefore, be the effector of hypoxic damage. This hypothesis, however, needs to be validated by further studies [31].

**Figure 1:**
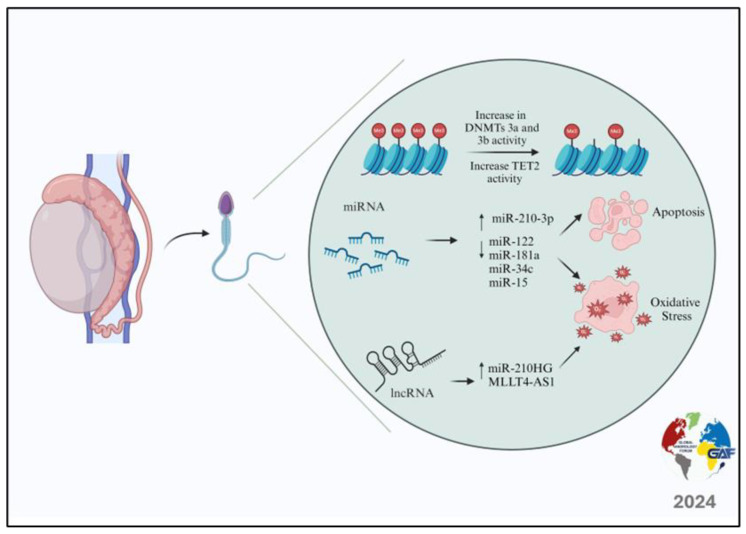
Epigenetic modifications in spermatozoa of patients with varicocele.

As shown by all of the studies discussed in this section, the expression profiles of non-coding RNAs appear to be fundamental in regulating the genesis of the damage induced by oxidative stress and apoptosis, two of the main mechanisms through which varicocele damages spermatogenesis (Figure 1).

## 3. Obesity, Metabolic Syndrome, and Epigenetic Modifications

Obesity is another disease that causes male infertility through several mechanisms, such as hyperinsulinemia, hyperleptinemia, chronic inflammation, and oxidative stress [32]. 

**Figure 2:**
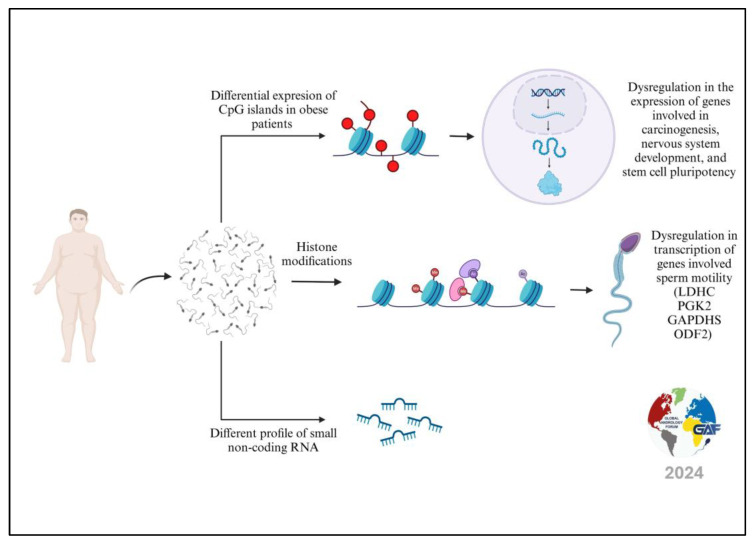
Epigenetic modifications caused by obesity in human spermatozoa.

Several studies have also highlighted the profound impact of obesity on epigenetic changes at the sperm level (Figure 2). However, most studies have focused on evaluating the possibility of the transmission of these epigenetic changes to offspring. At the same time, there is little information on the effects of these changes on patients’ fertility. Animal studies have shown that high-fat-fed obese mice develop the increased expression of the Dnmt1 and Dnmt3a genes at the testicular level, resulting in DNA hypermethylation [33]. Another study conducted in Wistar rats with genetic obesity confirmed the increased expression of the Dnmt1 and Dnmt3a genes at the testicular level, which caused DNA hypermethylation. At the same time, it showed the hypoexpression of the Dnmt1 gene and consequent sperm DNA hypomethylation in high-fat-fed obese mice [34]. Furthermore, the same authors demonstrated different patterns of testicular demethylation in the two groups of rats, probably due to the different distributions of the adipose tissue between the two groups. Other factors, such as oxidative stress, endocrine profiles, cytokine levels, and metabolite imbalances, may explain the different sperm DNA methylation profiles [35].

Human studies have also reported a significant difference in sperm DNA methylation in obese compared to normal-weight men [36,37], with differentially methylated CpGs for genes involved in transcriptional regulation and misregulation in cancer, nervous system development, and stem cell pluripotency [37]. While this evidence is significant regarding the possible risk of the transgenerational inheritance of these alterations, it reveals little about their impacts on sperm function and human fertility.

Regarding the methylation patterns of imprinted genes, it is known that the alteration of the methylation of some of these genes is associated with male fertility and spontaneous or post-assisted-reproductive-technique (ART) pregnancy rates [38]. A study on 23 obese patients and 44 normal-weight men showed, for example, that the methylation rate was significantly lower at the maternally expressed 3 (*MEG3*), Necdin (*NDN*), *SNRPN*, and sarcoglycan epsilon/paternally expressed 10 (*SGCE/PEG10*) DMRs and slightly higher at the *MEG3-IG* and *H19* DMRs in the spermatozoa of overweight/obese patients [39]. However, this evidence partially conflicts with the meta-analytic data present in the literature, which instead show a more significant percentage of *H19* gene hypomethylation in patients with oligozoospermia and with a history of recurrent pregnancy loss [6]. Similarly, higher rates of *SNRPN* gene methylation have been associated with male infertility [40]. However, it must be considered that, in the study on obese patients, the difference in the methylation profile was evaluated after adjustment for the patient’s fertility status [39]. Therefore, further studies are needed to better investigate the impact of obesity on the methylation levels of imprinted genes.

While the study of DNA methylation in the field of obesity has mainly focused on the risk of transgenerational inheritance, the role of histone modifications in contributing to obesity-induced infertility is more frequently investigated. Desphande and colleagues observed different changes in testicular histones of different cell types present in rats based on the presence of genetic or diet-induced obesity. Furthermore, an increase in core histones and a decrease in histone marks have been observed in spermatozoa, along with a sperm protamine deficiency, which alters the protamination process and, thus, chromatin integrity and the expression of specific genes related to sperm function [41]. In this sense, another study reported the dysregulation of genes involved in sperm motility in the testes of high-fat-fed obese mice. In particular, genes involved in cAMP/PKA-dependent signaling pathways, as well as the structural components of the sperm flagellum and ATP production pathways, appeared to be downregulated. Among these genes, lactate dehydrogenase C (*LDHC*), phosphoglycerate kinase 2 (*PGK2*), and glyceraldehyde-3-phosphate dehydrogenase spermatogenic (*GAPDHS*) encode three testis-specific glycolytic enzymes that are essential for sperm ATP production and are markers of the functional role of glycolysis in obesity-induced sperm motility abnormalities. Furthermore, the most downregulated gene appears to be the outer dense fiber of sperm tails 2 (*ODF2*), which encodes a major component of the outer dense fibers in the sperm tail. Moreover, the authors observed numerous histone modifications in the testes of obese mice, hypothesizing that these were responsible for the differences in genetic expression. However, the authors did not directly analyze this correlation [42]. To date, there is a lack of human studies that have evaluated the role of histone modifications in the pathogenesis of obesity-induced infertility.

Finally, regarding the expression of non-coding RNAs, animal studies have also highlighted the different expression profiles of miRNAs in a model of high-fat-fed obese mice compared to normal-weight mice [43]. However, even regarding this aspect, some studies have not evaluated the effects of these modifications in the pathogenesis of the damage induced to the spermatozoon.

## 4. Chronic Prostatitis and Epigenetic Modifications

Another highly prevalent condition associated with male infertility is chronic prostatitis. This condition affects 4.5–9% of men of reproductive age [44], with a recurrence rate of up to 50% with increasing age [45]. Meta-analytic studies have also observed its association with alterations in sperm parameters, a reduction in sperm concentration, motility, and a normal morphology [46]. Among the mechanisms proposed in the genesis of the damage induced to spermatozoa are the alteration of zinc levels and the appearance of anti-sperm antibodies [46]. However, there is little information on the direct epigenetic damage to spermatozoa. Indeed, there is lack of studies that have evaluated the impact of chronic prostatitis on histone modifications or the expression profiles of sperm non-coding RNAs. The only evidence to date has shown an altered protamination ratio in spermatozoa, which would explain the increased rate of DNA fragmentation in this category of patients [47]. As regards DNA methylation, only one study has analyzed the presence of possible methylation alterations at the seminal level, demonstrating the significant hypermethylation of the promoter of the *CXCR4* gene with a consequently reduced mRNA expression profile [48]. This chemokine, in turn, is involved in regulating the mast cell recall process; therefore, alterations in its pathway seem to be involved in the aberrant inflammatory responses of mast cells, which could explain chronic inflammation and sperm damage [48]. However, when the authors evaluated which subpopulations of cells in the seminal fluid were involved in this epigenetic alteration, they observed that the sperm DNA did not show methylation alterations; only the somatic cells were affected. Therefore, further studies are needed to understand whether prostatic inflammation can result in epigenetic changes in spermatozoa that are capable of altering their fertilizing capacity [48].

## 5. Lifestyle Habits and Epigenetic Modifications

### 5.1. Effects of Smoking Habits on Sperm Epigenetics

Cigarette smoking is another factor that can alter sperm parameters. A meta-analysis has shown that it is associated with reduced sperm counts and motility [49]. Several studies have examined the effects of cigarette smoking on sperm DNA methylation [50,51] and how it can influence sperm parameters [52,53]. A study of 14 heavy-smoking infertile patients, compared to 14 non-smoking fertile men, showed that there was a significant difference in DNA methylation at the level of several CpGs in amplicons related to the PGAM family member 5 (*PGAM5*), protein tyrosine phosphatase receptor type N2 (*PTPRN2*), and tyro3 protein tyrosine kinase (*TYRO3*) genes. This could potentially impair sperm development. Furthermore, the methylation of several CpGs was correlated with the sperm parameters. This study’s limitations include the comparison of an infertile and a fertile group, thus making it impossible to exclude the influence of other factors in the epigenetic changes described [52]. Another study with a similar design to this one reported that smoking patients had higher overall sperm DNA methylation levels than non-smokers. In addition, it showed a significant difference in the methylation levels of some CpGs of specific genes that thus were downregulated (aldehyde dehydrogenase 3 family member B2 (*ALDH3B2*), prostaglandin I2 receptor (*PTGIR*), and amyotrophic lateral sclerosis 2 chromosomal region candidate gene 12 (*ALS2CR12*)) or upregulated (prickle planar cell polarity protein 2 (*PRICKLE2*)). Finally, a negative correlation was observed between the methylation levels of differentially methylated CpGs in these genes and the alteration of the sperm parameters [53]. Interestingly, the same authors also evaluated the effects of waterpipe smoking compared with heavy cigarette smokers and non-smokers, confirming that cigarette smoking causes increased sperm DNA methylation, protamination deficits, and sperm DNA fragmentation, compared with non-smoking patients. These effects were even more pronounced in the waterpipe smokers, which therefore may be more harmful than cigarette smoking regarding sperm parameters, global DNA methylation, and the transcription of nuclear protein genes [54]. A direct association has been observed between the hypermethylation of the chromodomain helicase DNA-binding protein 5 (*CHD5*) gene, involved in the histone-to-protamine transition and DNA repair processes, and abnormal sperm parameters [55]. Overall, this evidence suggests that methylation changes in specific genes may contribute to the pathogenesis of cigarette-smoke-induced sperm damage.

At the sperm level in smokers, the differential expression of 28 miRNAs has been found, of which ten are involved in the regulation of cell proliferation, differentiation, and death pathways (mainly related to embryo development) and in pathways involved in diseases of the reproductive system. This suggests the contribution of non-coding RNA dysregulation in altering sperm parameters and embryo development in infertile smoking patients [56].

### 5.2. Effects of Alcohol on Sperm Epigenetics

Among the various substances of abuse, alcohol damages spermatogenesis in a dose-dependent manner [57]. Regarding the epigenetic effects of alcohol consumption at the sperm level, evidence suggests that it causes the hypermethylation of sperm DNA, as demonstrated by the increased methylation of the long interspersed nucleotide elements (LINE1) [58]. The methylation of specific imprinted genes could also be affected. In this regard, alcohol consumption has been associated with reduced methylation in some DMRs of the G protein subunit alpha S (*GNAS*) gene. In turn, the methylation levels of *GNAS* genes were negatively correlated with the sperm concentration, while the methylation levels of LINE1 were positively correlated with the follicle-stimulating hormone (FSH) levels, confirming the role of epigenetic modifications in alcohol-induced testicular and sperm damage [58]. Another study observed the hypomethylation of one specific DMR located in the CTCF binding site of the *H19* gene in the sperm DNA of men who consumed alcohol compared to non-consumers [59]. This finding is fascinating since the hypomethylation of the *H19* gene has been associated with oligozoospermia [6]. Therefore, the altered methylation of the *H19* gene could be another mechanism through which alcohol consumption impairs spermatogenesis.

However, little evidence, to date, exists for the role of alcohol in modifying sperm histones and the expression of non-coding RNA as a cause of impaired spermatogenesis. These changes are correlated with the possible transmission of anomalies to offspring, but it has not been demonstrated that they are associated with the alteration of sperm parameters [60,61].

### 5.3. Effects of Physical Activity on Sperm Epigenetics

Evidence suggests that recreational physical activity can positively affect sperm quality [62]. Epigenetic modifications could play a role in this beneficial action. A study on Wistar rats revealed that voluntary exercise improves spermatogenesis, partly by decreasing testicular oxidative stress and apoptosis by altering the miR-34a/SIRT1/p53 pathway [63]. Another study on mice examined the effects of exercise on high-fat-diet-fed obese mice. This study showed that obesity could impair sperm parameters such as motility and delay capacitation and acrosome reactions and that physical exercise could reverse these effects. An analysis of small non-coding RNAs in spermatogenic cells showed that three microRNAs were consistently upregulated and five were downregulated in round spermatids and spermatozoa samples from the epididymal region between obese and control mice. A pathway analysis highlighted that these changes in the expression of non-coding RNAs may play an essential role in the effects of obesity and physical exercise on male fertility and offspring development [64].

### 5.4. Effects of Dietary Habits on Sperm Epigenetics

Numerous randomized clinical trials and observational studies have explored the possible associations between diet and sperm quality by focusing on nutrients, dietary supplements, antioxidants, foods, and dietary patterns. An essential nutrient for epigenomic process metabolism is folic acid (folate or vitamin B9) via one-carbon metabolism [65]. Folate is essential for DNA stability, synthesis, and repair, preventing DNA oxidation by free radicals, and in the DNA methylation cycle [66,67]. Indeed, low paternal folate intake can change the sperm epigenome, resulting in worse sperm quality, reduced fertility, and congenital malformations. However, the evidence of this is more substantial in animal models than in humans [68].

Furthermore, a folate deficiency in the human diet can alter sperm DNA methylation in genes involved in development and metabolic processes [69]. However, not only a deficiency but also supplementation has been seen to have deleterious effects. Indeed, Ly and colleagues evaluated the effects on the offspring of female mice fed a diet with different types of folic acid supplementation. They showed that sperm of the offspring born from mice supplemented with folic acid seven times or 20 times higher than normal had significantly reduced sperm counts compared to controls born from mothers fed with a diet with average folic acid content. Moreover, sperm from mice born from mothers fed with folic acid at ten times higher levels than average showed greater variance in methylation across the *H19* imprinted gene, and increased variance at some sites within H19 was also found for the seven-fold folic acid deficiency group and 20-fold folic acid supplementation group. These data support the theory that the exposure of male mice to a high-folate-supplemented diet via their mothers results in decreased sperm counts and epigenetic alterations [70]. 

It has also been seen that the diet type can modify the testicular and sperm epigenetic profile. An animal study demonstrated that mice with high-fat-diet-induced obesity exhibited altered expression of 414 mRNAs and 11 miRNAs in the testes and a 25% reduction in global germ cell DNA methylation. Furthermore, alterations in sperm miRNA content were found [71]. However, the authors did not highlight the impact of these epigenetic changes on sperm and reproductive function. Still, they highlighted that these changes may be associated with alterations in the metabolic state of the following two generations [71].

### 5.5. Effects of Stress on Sperm Epigenetics

A study of 744 fertile men showed significantly lower sperm concentrations and motile sperm rates in patients experiencing more than two stressful life events, compared with patients with less than two stressful events [72]. Similarly, Zou and colleagues found a negative correlation between poor sperm parameters and stress of a psychosocial nature [73]. The mechanisms through which mental stress leads to a defect in testicular function are probably mediated by an increase in cortisol levels, which both exert a direct inhibitory effect on Leydig cells [74] but also suppress the gonadotropin-releasing hormone (GnRH) axis [75], with a consequent decline in testosterone levels and the impairment of spermatogenesis. Moreover, stress can create an imbalance in the seminal oxidative environment by reducing antioxidant enzymes, increasing radical oxygen species, and leading to sperm damage [76].

Oxidative stress can also cause epigenetic modifications, including sperm DNA methylation, histone modifications, and alterations in non-coding RNA expression [77,78]. In addition, an animal study has shown that oxidative stress in spermatozoa damages the DNA integrity and also affects the epigenetic reprogramming of the embryo. Specifically, oxidative stress in spermatozoa could impair the mechanisms proposed in DNA repair and DNA demethylation, thus altering the paternal genetic and epigenetic contribution to the developing embryo and thus impacting embryo development and quality [77].

### 5.6. Effects of Substance Abuse on Sperm Epigenetics

Substance abuse is the harmful use of psychoactive agents, including illicit drugs. Among them, cannabis is globally the most abused substance [79]. Signaling molecules interfere with the endogenous tone of the so-called endocannabinoids, which influence the quality of gametes [80].

The use of cannabis or delta-9-tetrahydrocannabinol (THC) in humans has been reported to decrease the sperm concentration, also leading to alterations in the sperm methylation of 183 cytosine guanine sites (CpG) related to 177 different genes. Similar findings were also reported in rats. An enrichment analysis showed the involvement of the differentially expressed genes in the hippocampal signaling and cancer pathways, both in humans and rats [81]. 

A transcriptomic study performed on cryopreserved spermatozoa obtained from bulls of known fertility and exposed to THC showed changes in differentially expressed genes depending on the concentrations of THC exposure. It was observed that 39 genes were differentially expressed after exposure to 0.032 µM THC, while 196 genes were differentially expressed if spermatozoa were incubated with a 10-fold higher THC concentration [82]. Furthermore, another study observed that THC exposure altered the expression of several fertility-associated miRNAs, including miR-346, miR-324, and miR-33b. In detail, the authors showed that the levels of these miRs decreased in spermatozoa exposed to 0.32 μM THC. However, the miR-34c levels were found to increase in spermatozoa exposed to 0.032 μM THC [83].

### 5.7. Effects of Exposure to Endocrine Disruptors on Sperm Epigenetics

Endocrine disruptor chemicals can influence the endocrine system by mimicking, blocking, or interfering with hormonal activity in humans and animals, leading to adverse health effects in humans and their offspring. These chemicals can modify sperm DNA methylation patterns, induce variable histone modifications, or alter miRNA regulation, leading to transcriptional changes that affect the reproductive system, even at very low doses [84].

Bisphenol A (BPA) is one of the compounds with endocrine disruptor activity that leads to reproductive system dysfunction [85]. Miao and colleagues studied the impact of BPA on sperm in factory workers exposed to it. Urine and semen samples were collected from the exposed workers and a control group to measure the individual levels of BPA and perform a LINE-1 methylation analysis, respectively. A multivariate analysis adjusted for age, education, tobacco smoking, and alcohol consumption showed a significant decrease in the methylation level of LINE-1, a marker of the global methylation status, in the BPA-exposed group [86]. Similarly, a genome-wide DNA hydroxymethylation study showed a significant increase in 5-hydroxymethylcytosine (5hmc) levels (19.37%) in BPA-exposed men in 72.69% of the genome regions located in the intergenic and intron regions harboring 5hmc compared to controls. Furthermore, the authors showed an association between the alteration in DNA hydroxymethylation induced by BPA and three different types of H3 histone methylation (H3K4me2, H3K4me3, or H3K27me3) in sperm, suggesting that the effects of BPA on the DNA hydroxymethylation of genes is partly dependent on the trimethylation of H3 in human spermatogenesis [87]. Therefore, these epigenetic changes induced by BPA could be another mechanism by which this substance can determine sperm toxicity [87].

## 6. Focus on the Possibility of Transmitting Epigenetic Changes to Offspring (Transgenerational Inheritance) and Their Health Consequences

The study of epigenetics has attracted growing interest over the years due to the possibility of the transgenerational transmission of these changes and their influence on offspring health. In this sense, although most epigenetic modifications are erased in germ cells and at the time of fertilization, some alterations, such as imprinted gene methylation changes, histone modifications, or non-coding RNAs, can be transmitted and thus modulate gene expression [88]. For example, changes in the sperm epigenome and small non-coding RNAs in obese patients have been found to have a significant impact on offspring health [89]. In detail, a study on a mouse model has shown that, in the liver cells of the daughters of obese male mice, there is the hypomethylation of the imprinting control region (ICR) of the h19/igf2 gene, which is also present in the father’s spermatozoa. This would suggest that epigenetic changes in germ cells contribute to this father–child transmission [90]. The authors also demonstrated that the resulting overexpression of the H19 transcript upregulates gluconeogenesis, thus suggesting that paternal obesity can alter gluconeogenesis and, therefore, the metabolism of the offspring through the dysregulation of the methylation of this gene [90].

DNA methylation and histone modifications are involved in this process of transgenerational inheritance. For example, the overexpression of sperm histone H3 lysine 4 tri-methylation (H3K4me3) is involved in mice, in regulating the gene expression of several genes implicated in offspring metabolic, inflammatory, and developmental processes [91]. The risk of transmitting alterations to offspring may be associated not only with obesity but also with exposure to toxic substances. For example, the significant hypermethylation of sperm DNA and the DMR of the protein delta homolog 1 (*DLK1*) gene was observed in male smokers. The authors then observed the same alteration in a mouse model, with the overexpression of the dlk1 gene in the livers of the male offspring of these mice, which in turn was correlated with an increase in hepatic fat accumulation and an altered response to the glucose load test. This suggests that epigenetic alterations induced by cigarette smoking may also be transmitted to offspring by altering their metabolic function [92]. The type of diet, as highlighted above, is also likely to impact the future health of offspring profoundly [71]. Finally, not only the general health of the offspring but also their future fertility may be affected by this mechanism. Indeed, in mice, it has been observed that exposure to toxic substances can lead to alterations in testicular function that can be passed on to unexposed offspring. The authors showed that the modifications in the methylation profile, non-coding RNA, and messenger RNA that were observed in the Sertoli cells of exposed mice were also present in the Sertoli cells of subsequent generations, suggesting that changes in the epigenetic profile may be responsible for fertility damage. This could help to explain the progressive decline in male fertility that has been witnessed in recent decades [93].

These are just a few examples of the numerous works in the literature that demonstrate the possibility of the risk of transgenerational inheritance. 

## 7. Future Directions

From what has been discussed so far, it is clear that there is great interest in studying the influence of the environment and lifestyle factors on epigenetics and how this can influence reproductive function and offspring health. However, a deeper understanding of the ways in which epigenetic modifications damage human spermatogenesis is necessary, as this is often missing in the studies examined in this review. This study therefore highlights how future research should be better directed towards the analysis of the molecular mechanisms through which these epigenetic modifications interfere with spermatogenesis.

The risk of transgenerational inheritance is much more widely studied. However, even in this case, if animal studies reach more solid conclusions on the risk of transgenerational transmission, human studies report conflicting results. One example is the study conducted by the Pregnancy and Childhood Epigenetics (PACE) consortium, which did not find any correlation between the paternal body mass index and changes in global methylation and the imprinted regions of DNA in offspring [94]. Therefore, further studies are certainly needed to better clarify the role of the transgenerational inheritance of epigenetic modifications in human offspring. In this regard, in the literature, there is a lack of human studies that evaluate the effects of epigenetic changes induced by non-genetic forms of male infertility, not only in the offspring of the affected population but also in the subsequent generation. Only such studies would allow us to understand whether an epigenetic modification transmitted to offspring is also able to perpetuate itself in future generations, thus becoming a new genetic trait.

## 8. Conclusions

**Figure 3:**
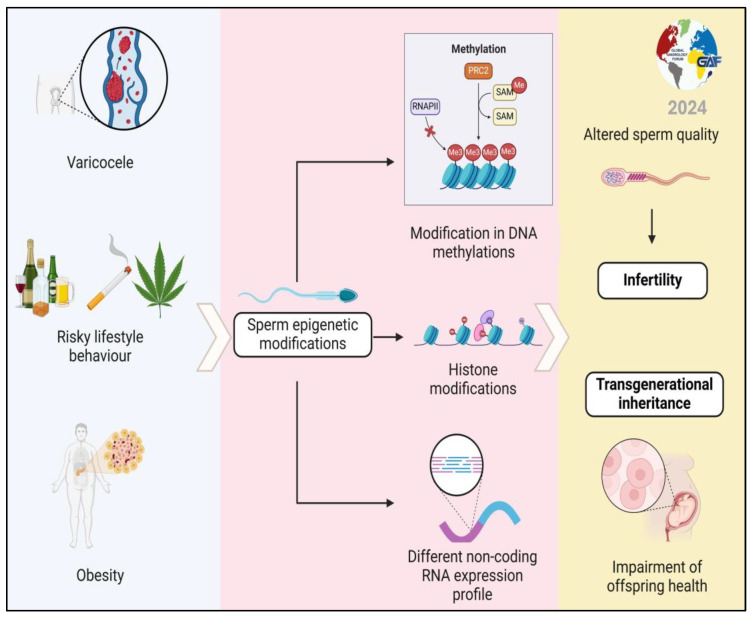
Sperm epigenetic modifications induced by non-genetic causes of infertility can alter fertility and increase the risk of transmitting abnormalities to offspring.

In conclusion, this review highlights how even the causes of infertility classically considered as a non-genetic etiology can modify epigenetics and, therefore, the regulation of gene expression, which contributes to damage to reproductive function (Figure 3). This evidence also has another important implication. Considering the risk of the transgenerational inheritance of epigenetic modifications, treating these non-genetic conditions becomes fundamental not only for fertility purposes but also for the health of offspring (Figure 3). 

In this sense, identifying epigenetic modifications at the sperm level that are potentially transmissible to the offspring of patients with varicocele could become a further criterion for the selection of patients to undergo varicocele repair. Likewise, considering the epigenetic effects of an unhealthy lifestyle and exposure to toxic substances enables us to understand the fundamental role of clinicians in educating patients seeking to improve their fertility and the importance of correcting risk factors for the well-being not only of patients but also of future generations.
